# Face masks negatively skew theory of mind judgements

**DOI:** 10.1038/s41598-023-31680-y

**Published:** 2023-03-27

**Authors:** Héctor Leos, Ian Gold, Fernanda Pérez-Gay Juárez

**Affiliations:** 1 Interfaculty Program of Cognitive Science, McGill University, Montreal, H3A 0G4 Canada; 2 Departments of Philosophy and Psychiatry, McGill University, Montreal, H3A 0G4 Canada

**Keywords:** Human behaviour, Emotion, Social behaviour

## Abstract

Face masks obscure a significant portion of the face, reducing the amount of information available to gauge the mental states of others—that is, to exercise the Theory of Mind (ToM) capacity. In three experiments, we assessed the effect of face masks on ToM judgements, measuring recognition accuracy, perceived valence, and perceived arousal in various sets of facial expressions comprising 45 different mental states. Significant effects of face masks were found in all three variables. Judgements of all expressions are less accurate when masked, but, while judgements of negative expressions do not show consistent changes in valence or arousal, positive expressions are perceived to be less positive and less intense. In addition, we identified face muscles associated with changes in perceived valence and arousal, shedding light on the mechanisms through which masks impact ToM judgements, which might be relevant for mitigation strategies. We discuss the implications of these findings in the context of the recent pandemic.

## Introduction

Social life around the world has been profoundly impacted by the COVID-19 pandemic^1^, a disease spread mainly by close social contact^2^. For much of the last three years, in-person social exchanges have been restricted by public health measures and contracted to virtual interactions^3^. Moreover, fear of contagion and proximity to others has been a source of tension and anxiety in actual face-to-face gatherings^4^. But ultimately, among the most pervasive obstacles to social exchanges has been the use of face masks; in covering the face, a mask obscures facial expressions and seems to make it more difficult to tell what someone is thinking or feeling—an ability known as Theory of Mind (ToM)^5^, mindreading, or mentalizing. ToM is widely regarded as essential to social exchange and is thought to underlie the capacity for empathy^6^, compassion^7^, and other forms of prosocial responsiveness^8^. Impairments of ToM have been linked to various neuropsychiatric disorders including those in the autistic spectrum^9,10^, and schizophrenia^11–13^, partially mediating impaired social decision-making in the latter^14^. While originally conceptualized as a reflective, effortful task requiring higher-order processes such as language and executive functions^15,16^, ToM in fact appears to depend on a collection of neurocognitive functions including the more automatic, perceptual processes through which we infer mental states from bodily motion^17^, including gaze direction and facial gestures^18^, in a similar way to which we recognize basic emotions^19^. By obscuring much of the face, masks are obstacles to the detection of facial expressions and, as a result, to the contribution faces make to mental state judgements.

Previous research studies have already reported that face masks hinder face recognition abilities by disrupting holistic processing (i.e. perceiving faces as undifferentiated units instead of attending to their individual features), as suggested by a reduced face-inversion effect^20,21^. Furthermore, a series of studies has assessed the impact of face masks on emotional recognition in different age groups, reporting changes in recognition accuracy^22–26^, and reduced confidence in one’s emotion recognition judgements^22^. Masks also impact social and moral judgements such as those concerning trustworthiness, likability and closeness^24^, and desired physical distance from people^27^. These studies, however, have limited their investigations to the six basic emotions (fear, anger, joy, sadness, disgust, and surprise) or to some modification of them. While the six basic emotions are profoundly important, they represent only a very small subset of the range of mental states identified by agents seeking to understand and connect with others. Critically, ToM also targets non-emotional mental states as well as states with propositional content including beliefs, desires, and intentions. Accurate identification of these states provides resources for explaining and predicting the behavior of others^28^, both of which are deeply valuable for social exchanges.

To better understand the impact of masks on ToM, we carried out two experiments that included a wider range of mental states beyond the six basic emotions. More specifically, the experiments made use of 45 pictures of faces depicting positive, neutral, and negative mental states, grouped in three sets (unmasked faces, faces wearing a KN95 face mask, and faces wearing a neck gaiter). In the first experiment we examined mental state recognition, by asking participants to choose an appropriate mental state term for each stimulus. In the second experiment, to further qualify the effect of masks, we asked participants to rate the valence and arousal of the facial expressions in an affect grid^29^. The addition of a dimensional measure provides a more fine-grained picture of the consequences of mask-wearing on ToM^30^. To corroborate our findings, we repeated the second experiment with a more diverse set of stimuli. Finally, we identified the face muscles that were most strongly associated with changes in perceived valence and arousal. We discuss the implications of our findings in the context of the pandemic, considering in particular the potential consequences of ToM on psychological well-being.

## Results

### Mental state recognition

**Figure 1:**
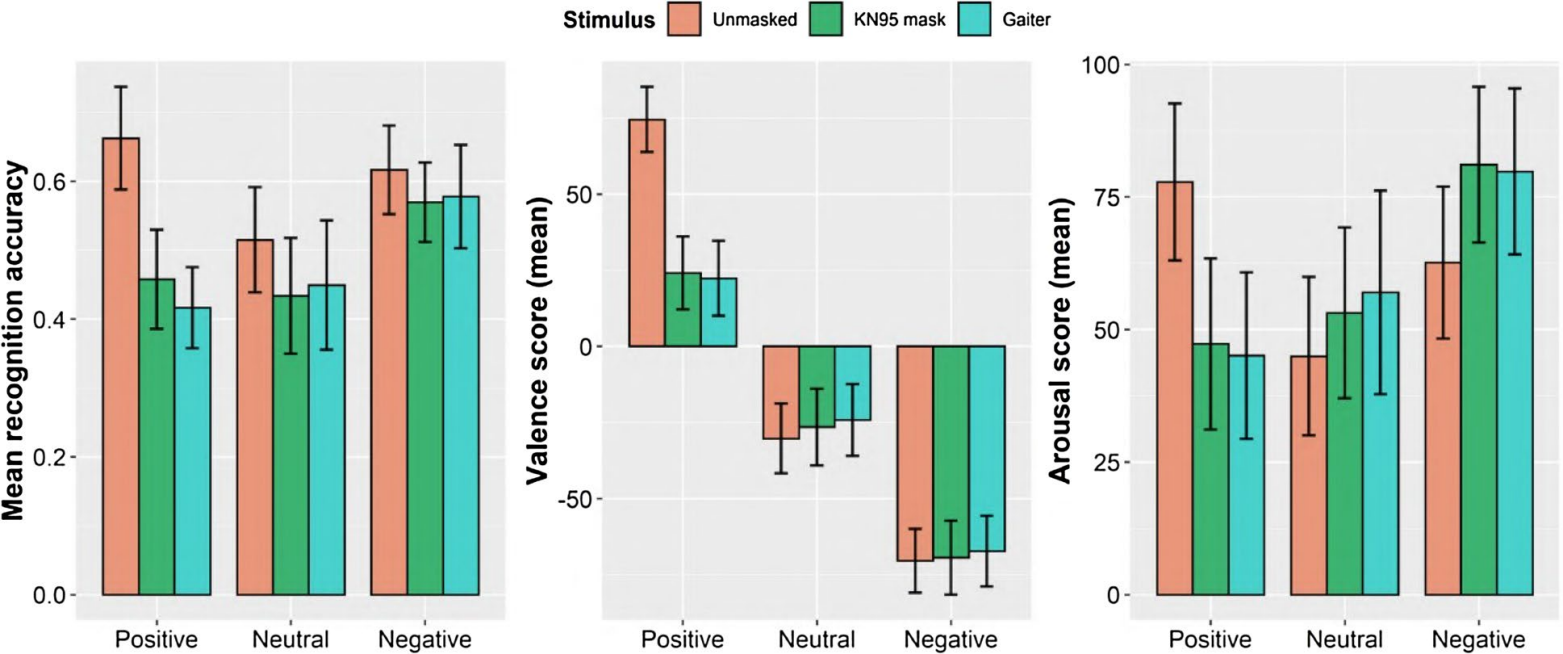
Results of Experiments 1 and 2. Left: Average recognition accuracy for positive, neutral, and negative mental states across the three conditions. (Note that the guess rate in a four-alternative forced choice paradigm is 25%.) Middle: Average perceived valence scores showing a significant decrease in valence between the unmasked and the two covering conditions for positive mental states (but not for the neutral and negative ones). Right: Average perceived arousal scores showing a significant decrease in perceived arousal for the positive expressions and a significant increase for neutral and negative ones. Error bars represent standard deviation of the mean.

**Figure 2:**
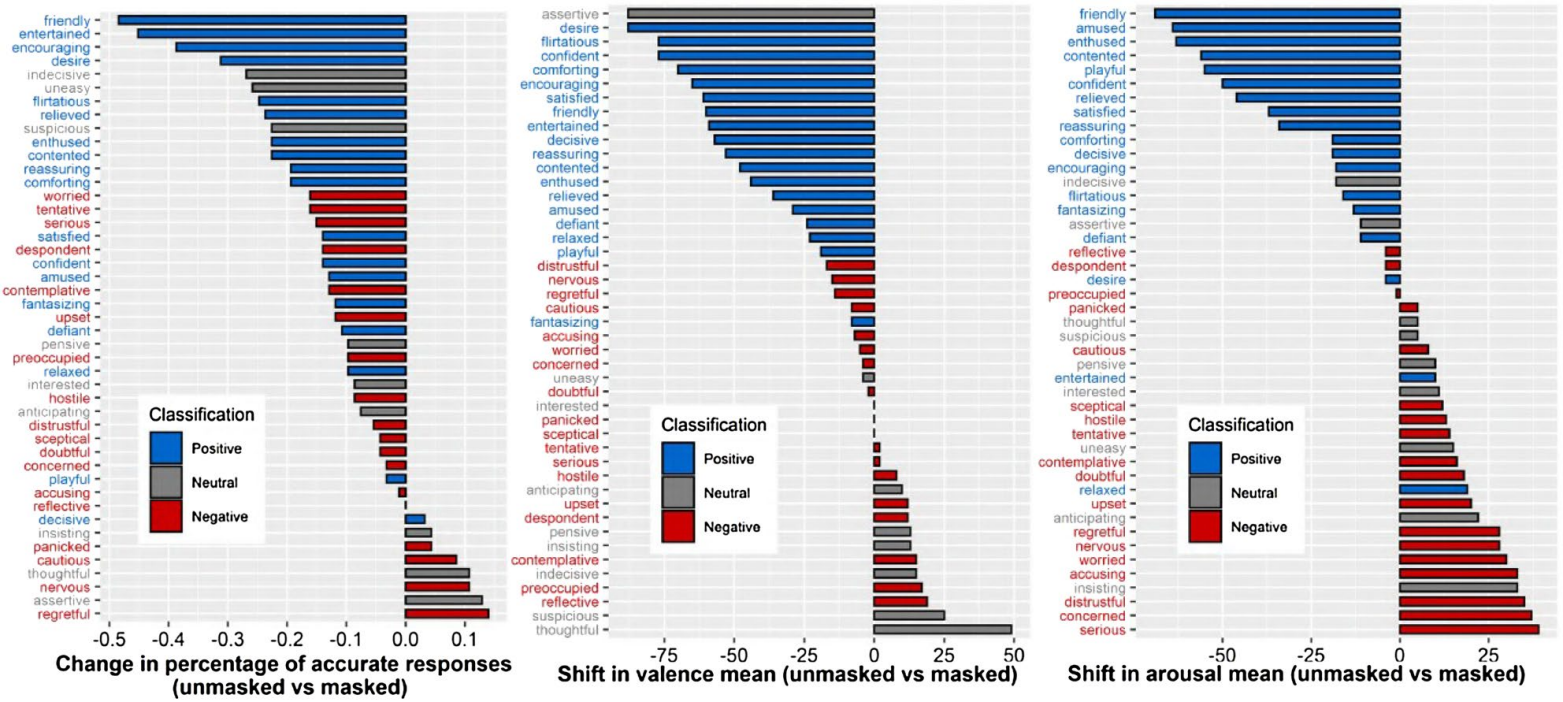
Changes in means of recognition accuracy, perceived valence and perceived arousal between the unmasked and the KN95 conditions for each mental state. Left: Masking impairs the recognition of most facial expressions. Middle: All the mental states previously classified as positive undergo a negative shift in valence. Right: Perceived arousal decreases for positive mental states but increases for negative mental states.

In Experiment 1, 93 participants were asked to identify the mental state associated with each of 45 facial expressions by choosing the appropriate mental state term in a four-alternative forced-choice paradigm. A Friedman test was conducted to compare the average recognition accuracy in each of the mental state categories (positive, neutral, and negative), between three conditions (unmasked, KN95 mask, and gaiter). We found a significant effect of condition in recognition accuracy in positive mental states (*χ*^2^(2) = 96.68, *p* < 0.0001, *W* = 0.52,*CI* = [0.42, 0.64]), as well as in neutral ones (*χ*^2^(2) = 9.22, *p* = 0.01,*W* = 0.05,*CI* = [0.01, 0.13]). No significant effect was found for negative mental states (*χ*^2^(2) = 5.54, *p* = 0.063,*W* = 0.03,*CI* = [0.004, 0.1]). These results are depicted in Fig. 1 (left). Post-hoc tests revealed significant differences in accuracy between the unmasked and the two covering conditions (KN95 and gaiter) in all categories, with the largest effect sizes found for positive expressions (see Table 1). When comparing accuracy in the KN95 and the gaiter conditions, a significant effect was found only for the positive mental states. Figure 2 (left) depicts changes in mean recognition accuracy for individual mental states when comparing responses for the unmasked and the KN95 condition, highlighting the negative effect of masking on accurate recognition of most positive expressions.

**Table 1:** Friedman tests post-hoc analysis for Experiments 1 and 2: Wilcoxon signed-rank tests (Bonferroni corrected) were used to compare pairs of the three conditions.

Variable	Condition	Tested pairs	*Z*	Effect size	95% CI
Recognition accuracy (Experiment 1)	Positive	Unmasked-KN95	7.548***	0.78	[0.70, 0.84]
		Unmasked-Gaiter	7.908***	0.82	[0.76, 0.86]
		Gaiter-KN95	− 2.367*	0.25	[0.04, 0.44]
	Neutral	Unmasked-KN95	3.038**	0.31	[0.13, 0.50]
		Unmasked-Gaiter	2.894**	0.30	[0.10, 0.48]
		Gaiter-KN95	0.545	0.06	[0.004, 0.27]
	Negative	Unmasked-KN95	2.994**	0.31	[0.12, 0.49]
		Unmasked-Gaiter	2.068*	0.21	[0.02, 0.39]
		Gaiter-KN95	0.229	0.02	[0.002, 0.23]
Valence score (Experiment 2)	Positive	Unmasked-KN95	8.294***	0.86	[0.84, 0.87]
		Unmasked-Gaiter	8.332***	0.86	[0.85, 0.87]
		Gaiter-KN95	− 0.529	0.05	[0.004, 0.26]
	Neutral	Unmasked-KN95	− 0.902	0.09	[0.007, 0.30]
		Unmasked-Gaiter	− 1.510	0.16	[0.01, 0.35]
		Gaiter-KN95	1.013	0.11	[0.004, 0.31]
	Negative	Unmasked-KN95	− 0.130	0.01	[0.004, 0.24]
		Unmasked-Gaiter	− 1.151	0.12	[0.006, 0.32]
		Gaiter-KN95	1.151	0.12	[0.008, 0.31]
Arousal score (Experiment 2)	Positive	Unmasked-KN95	6.462***	0.67	[0.56, 0.77]
		Unmasked-Gaiter	6.951***	0.72	[0.61, 0.80]
		Gaiter-KN95	− 0.895	0.09	[0.005, 0.31]
	Neutral	Unmasked-KN95	− 2.669**	0.28	[0.09, 0.47]
		Unmasked-Gaiter	− 3.197**	0.33	[0.14, 0.52]
		Gaiter-KN95	1.090	0.11	[0.008, 0.30]
	Negative	Unmasked-KN95	− 5.284***	0.55	[0.40, 0.69]
		Unmasked-Gaiter	− 4.920***	0.51	[0.34, 0.66]
		Gaiter-KN95	− 0.274	0.03	[0.004, 0.24]

Effect sizes were calculated by diving the *Z* value by the square root of *N*. Note that the groups in Experiments 1 and 2 were independent, and each was of size *N* = 93.

***p < 0.001, **p < 0.01, *p < 0.05.

### Perceived valence and arousal

In Experiment 2, 93 participants (different from those in Experiment 1) assessed each mental state on a grid representing valence and arousal. Friedman tests were conducted to compare perceived valence and perceived arousal for each mental state category between the unmasked, the KN95 and the gaiter conditions. We found a significant effect of condition on perceived valence for positive expressions (*χ*^2^(2) = 119.83, *p* < 0.0001,*W* = 0.64, *CI* = [0.56, 0.73]), but not for neutral or negative ones (Neutral, *χ*^2^(2) = 0.21, *p* = 0.90,*W* = 0.001,*CI* = [< 0.001, 0.04]; Negative, *χ*^2^(2) = 2.00, *p* = 0.37, *W* = 0.01, *CI* = [< 0.001, 0.07]). These results are shown in Fig. 1 (middle). Post-hoc tests (see Table 1) revealed that differences in perceived valence between the unmasked condition and both the KN95 and gaiter conditions were significant for positive expressions and had a large effect size (0.86 in both unmasked-KN95 and unmasked-gaiter). No significant differences in valence were found between the KN95 mask and the gaiter conditions for any mental state category.

Significant effects of covering condition on perceived arousal were found in all valence categories (Positive, *χ*^2^(2) = 46.22, *p* < 0.0001, *W* = 0.25,*CI* = [0.14, 0.39]; Neutral, *χ*^2^(2) = 8.86, *p* = 0.012,*W* = 0.05,*CI* = [0.008, 0.14]; Negative, *χ*^2^(2) = 34.13, *p* < 0.0001,*W* = 0.18,*CI* = [0.09, 0.32]). Post-hoc tests (see Table 1) revealed that the directionality of this change varied depending on the valence category: while positive mental states showed a significant decrease in perceived arousal between the unmasked and the two covering conditions, neutral and negative mental states showed a significant increase in perceived arousal. (Note that the neutral category contains slightly negative mental states, see the Methods section.) These results suggest that, when using face masks, positive facial expressions are perceived as less aroused, while negative expressions are perceived as more aroused. For a visual depiction of the shifts in valence and arousal scores (unmasked vs KN95) across individual mental states, see Fig. 2 (middle and right).

**Figure 3:**
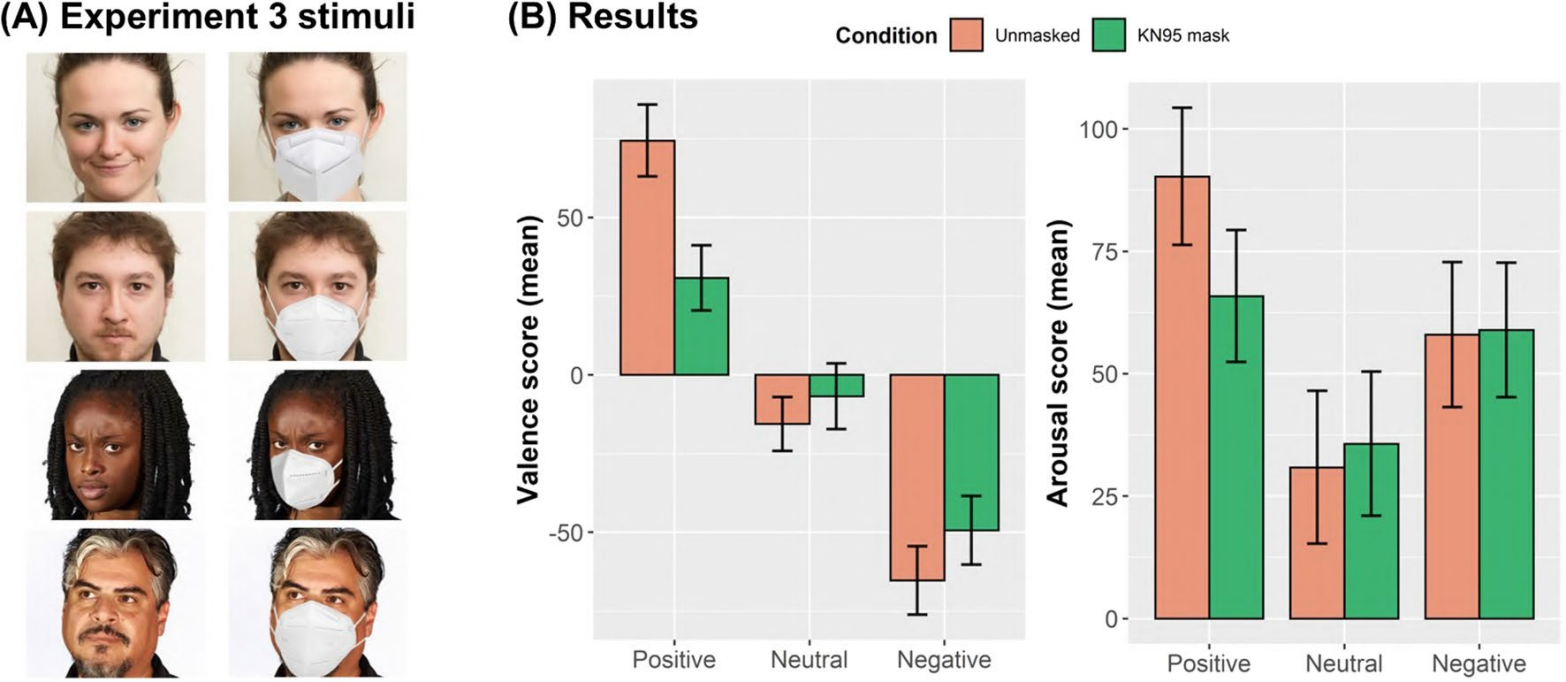
Experiment 3 stimuli and results. (**A**) The same 45 mental states used in Experiments 1 and 2 were selected for each of four different actors, for a total of 180 unmasked stimuli and 180 masked stimuli. (**B**) When combining all actors together, mean valence scores decreased significantly in valence when comparing unmasked and masked faces for positive mental states, but increased significantly in valence for the neutral and negative categories. Furthermore, there was a significant decrease of perceived arousal scores for positive mental states. Error bars represent standard deviation of the mean.

In Experiment 3 we had an additional group of 100 participants using the same affect grid as in Experiment 2 to rate the facial expressions of four different actors, with the goal of addressing whether the results would extend across exemplars (to faces of other people). Since only the unmasked and the KN95 mask conditions were included, Wilcoxon signed-rank tests were used to assess differences in valence and arousal scores. As in Experiment 2, we found a significant decrease in both valence and arousal for positive mental states (Valence, Z = 8.671, p < 0.0001, ES = 0.87,CI = [0.86, 0.87]; Arousal, Z = 6.916, p < 0.0001, ES = 0.69,CI = [0.58, 0.78]). We also found a significant increase in valence for neutral and negative mental states (Neutral, Z = 3.617, p < 0.001, ES = 0.36,CI = [0.19, 0.53]; Negative, Z = 7.128, p < 0.0001, ES = 0.71,CI = [0.62, 0.79]), but no significant change in perceived arousal (Neutral, Z = 1.960, p = 0.050, ES = 0.20,CI = [0.02, 0.38]; Negative, Z = 0.254, p = 0.8, ES = 0.025,CI = [0.003, 0.24]). The results are depicted in Fig. 3B. The bar plots for each of the four actors are shown in Figures S1 and S2 in the Supplementary Material (Table S1 displays the corresponding statistical analyses).

### Predictive facial features

Noldus FaceReader software was used to obtain the activation scores of the Facial Action Coding System (FACS) action units (AU)—which correspond to the components of muscle movements as well as head movements—in each of the 45 facial expressions of the female actor whose pictures were presented in Experiments 1 and 2. We then selected nine AUs that had been previously found to be associated with positive and negative facial expressions^31^ and ran six multiple linear regressions using these AUs as independent variables. The mean scores in valence and arousal for the unmasked and KN95 conditions, as well as for the change between these two conditions, were used as the dependent variables. The results can be found in Table 2. The model for the difference in valence obtained an adjusted *R*^2^ of 0.77, suggesting that the chosen AUs explain up to 77% of the variance in our model. In the case of the difference in arousal scores, the model obtained an adjusted *R*^2^ of 0.65. All of our models were statistically significant.

**Table 2:** Multiple linear regression results. The independent variables (rows) are FACS AUs that have been previously found to be associated with either positive or negative valence.

	Valence			Arousal		
	Unmasked	KN95	Difference	Unmasked	KN95	Difference
Head pitch	0.21***	0.50***	0.30**	0.06	− 0.06	− 0.16
AU 1 Inner brow raiser	− 0.06	− 0.17**	− 0.12	0.34***	0.32**	< 0.001
AU 4 brow lowerer	− 0.20**	− 0.44***	− 0.24*	0.41***	0.41**	0.03
AU 5 upper lid raiser	− 0.02	0.02	0.07	0.37***	0.47***	0.16
AU 6 cheek raiser	0.17*	0.40***	0.23	0.31**	0.10	− 0.25
AU 10 upper lip raiser	− 0.02	0.01	0.05	0.23**	0.23*	0.02
AU 12 lip corner puller	0.74***	0.31**	− 1.03***	0.54***	0.13	− 0.50**
AU 25 lips part	− 0.12	− 0.28**	− 0.16	0.27*	0.13	− 0.16
*R*^2^	0.93	0.90	0.81	0.87	0.74	0.72
Adjusted *R*^2^	0.91	0.88	0.77	0.84	0.69	0.65
*F*-statistic	59.63*** df (8, 36)	41.11*** df (8, 36)	19.15*** df (8, 36)	29.30*** df (8, 36)	13.01*** df(8,36)	11.43*** df (8, 36)

Six different models were run, each predicting one of the dependent variables (columns): the mean scores in valence and arousal for the unmasked and the KN95 conditions, as well as for the difference between the two conditions.

***p < 0.001, **p < 0.01, *p < 0.05.

**Figure 4:**
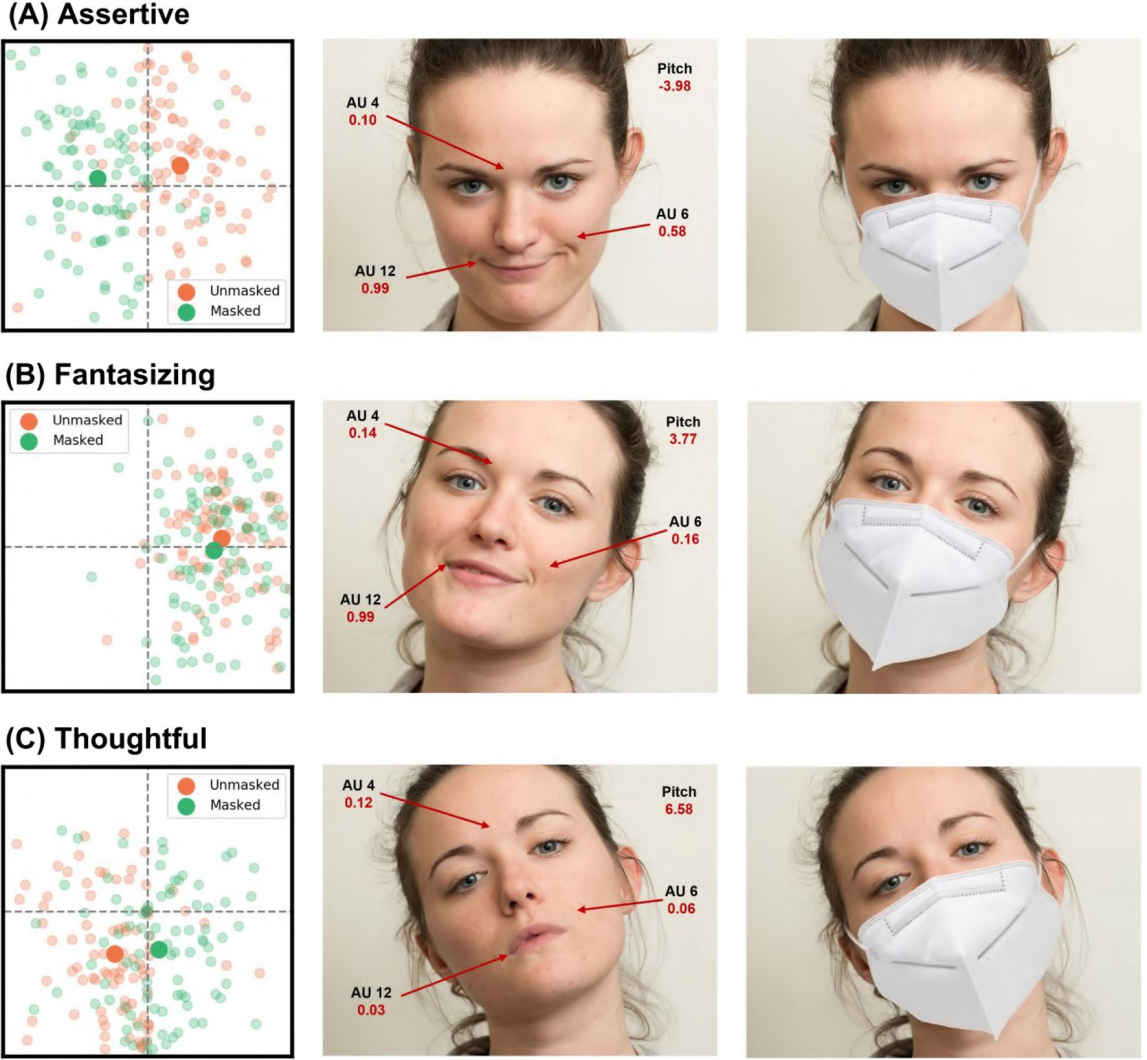
Comparisons of unmasked vs KN95 valence scores (x-axis) for three mental states that showed different degrees of valence changes. Lighter datapoints are individual ratings, and darker datapoints are the mean ratings in the 2D grid. Nodding forward (i.e. negative pitch) makes positive expressions appear more negative in the KN95 condition (**A**). On the contrary, nodding backward (i.e. positive pitch) helps preserve the positive valence (**B**), and renders negative mental states more positive. (**C**). Other predictors of valence are frowning (AU 4), raising the cheeks (AU 6) and smiling (AU 12). Face images were obtained with permission from the public McGill Face Database^32^.

**Figure 5:**
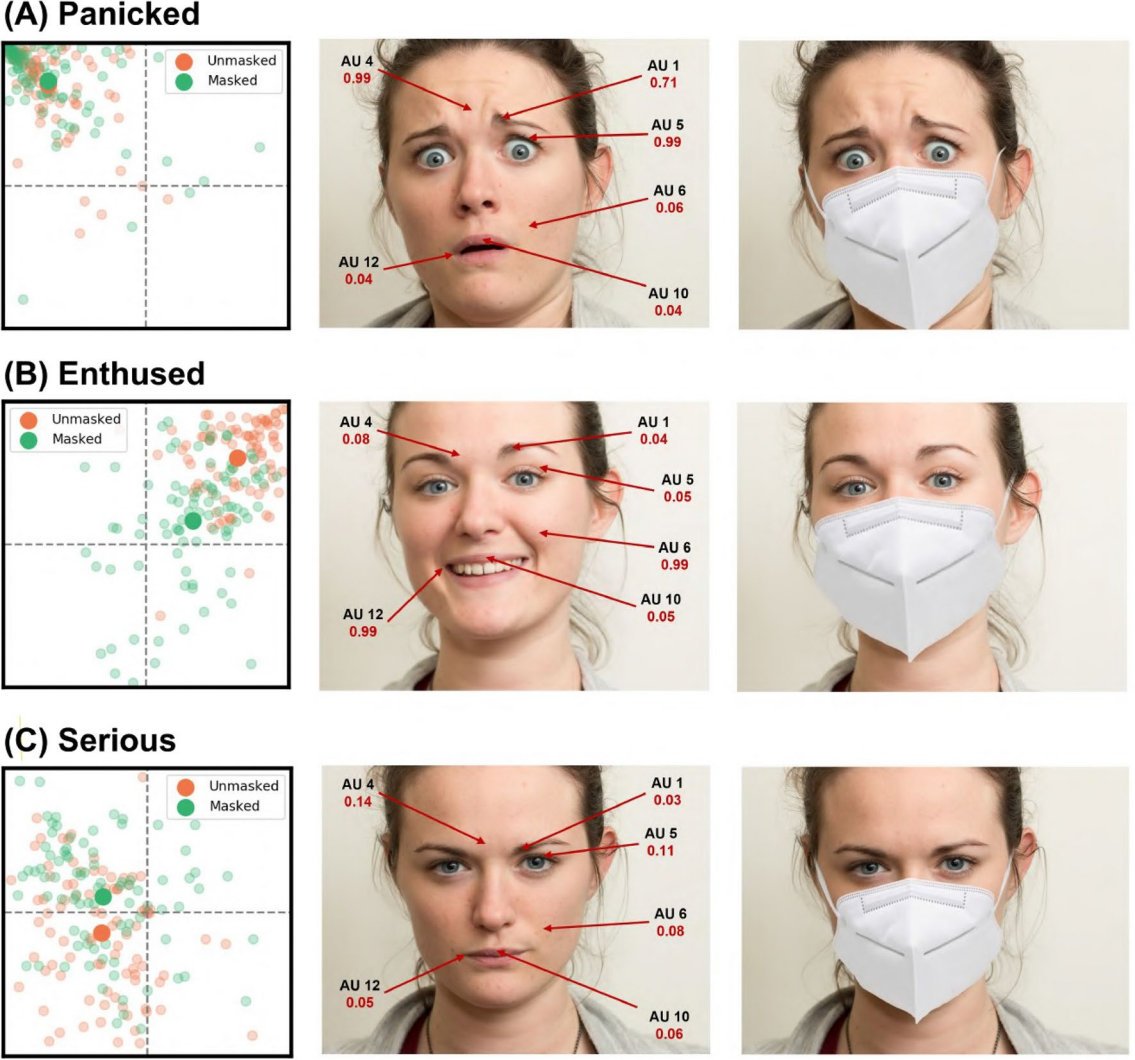
Comparisons of unmasked vs KN95 arousal scores (y-axis) for three mental states with variable degrees of arousal changes. Lighter datapoints are individual ratings, and darker datapoints are the mean ratings in the 2D grid. Raising the inner brow (AU 1), frowning (AU 4), and raising the upper eye lids (AU 5) are strong predictors of arousal in both conditions (**A**). Other predictors include raising the cheeks (AU 6) and the upper lip (AU 10), and smiling (AU 12), all of which are obscured by face masks, resulting in lower arousal scores (**B**). Masking was also found to intensify negative facial expressions (**C**). Face images were obtained with permission from the public McGill Face Database^32^.

The AU that was most strongly associated with negative changes in both valence and arousal was the Lip Corner Puller (AU 12). In contrast, the variable most associated with a positive change in valence when using the face mask was a positive value of the Head Pitch (i.e. nodding backwards). Three mental states along with their corresponding scores for perceived valence in both the unmasked and the KN95 condition are shown in Fig. 4, illustrating the interaction between smiling (AU 12) and nodding in face masking. In the context of arousal, Inner Brow Raiser (AU 1), Brow Lowerer (AU 4), and Upper Lid Raiser (AU 5) remain strong predictors of arousal when wearing a face mask (refer to columns 4 and 5 in Table 2). Figure 5 shows some examples of facial expressions with their corresponding scores for perceived arousal, illustrating the role of the relevant AUs in score differences between the unmasked and the KN95 conditions.

Cheek Raiser (AU 6) was positively correlated with both Upper Lip Raiser (AU 10) and Lips Part (AU 25) (Figure S3 in the Supplementary Material displays the correlation matrix for our AUs). We therefore ran additional linear regression models without this AU. This resulted in a decrease of the adjusted *R*^2^ from 0.77 to 0.75 for changes in perceived valence and changed the standardized coefficient of Head Pitch to *β* = 0.25 and that of the Lip Corner Puller to *β* = 0.94. In the case of the difference in arousal scores, the model without Cheek Raiser obtained an adjusted *R*^2^ of 0.64, and it increased the standardized coefficient of Lip Corner Puller to *β* =  *− *0.59. Considering the Brow Lowerer was also negatively correlated with the Lip Corner Puller, we ran a third series of models without this AU. This changed the predictive value of the Brow Lowerer for the change in perceived arousal, making it significant (*β* = 0.29, *p* = 0*.*029), with an adjusted *R*^2^ of 0.57 for this model. The linear regression models with higher adjusted *R*^2^’s were the ones including all of our a priori chosen AUs, shown in Table 2.

## Discussion

Face masks have been a crucial tool in fighting the COVID-19 pandemic and have also been a locus of considerable social and political division in many countries. Given the ubiquity and salience of masks, a good deal of recent research has been devoted to studying their effect on social interactions^20–26^. This research is essential both to understand the role that masks have played in recent years and to better prepare for the impact they may have in the future. The findings we report here support the view that masking has a measurable effect on social cognition, in a direction that is meaningful and practically important. Overall, we find that masks not only impair the ability to recognize facial expressions—particularly the ones representing positively-valenced mental states—but also tend to skew the valence and arousal associated with them in a negative direction.

Considering Experiments 1 and 2 together, we find (a) a significant decrease in recognition accuracy, with positive mental states significantly more affected than neutral or negative states; (b) a shift in perceived valence towards negativity in positive states; and (c) an interaction between arousal and valence in which masking renders positive expressions less intense, and negative and neutral expressions more intense. Our results for the gaiter condition suggest that these effects derive not from the masks per se, but rather from the occlusion of the lower half of the face. In Experiment 3, we used a more diverse set of actors to explore whether the effects would extend across other faces. We included two more male actors (one white, one latino) and one more black female actor, and measured valence and arousal judgements for masked and unmasked expressions. In this third experiment, we corroborated our results for the judgements of positive mental states, which were perceived as significantly less positive and significantly less intense. We also corroborated the increase in perceived arousal for expressions of neutral mental states. However, the initial effect found for negative mental state expressions, which were perceived as equally negative but more intense, was not replicated. A comparison of the results for each individual actor (Figures S1 and S2; Table S1) suggests that, instead of being a product of the individual expressive pattern of the female actor in Experiment 2, what might be driving this effect is a possible gender bias: whereas there is a significant increase in valence for negative mental states in the two male actors when comparing the unmasked and the masked conditions, there is no significant change in perceived valence for the two female actors. When looking at positive expressions, the decrease in positivity is also larger for female actors than for male actors, suggesting that the negative bias is stronger when mindreading female masked faces. This opens up new possibilities regarding gender differences in mindreading with face masks which should be further investigated in future research using a larger set of actors.

In the context of previous research of masked face expressions, the overall decrease in recognition accuracy of facial expressions reported here confirms earlier findings. However, our findings regarding which expressions are most affected do not. Carbon^22^ reports that both negative emotions (anger, sadness) and a positive emotion (happiness) were mistaken for a neutral state, and McCrackin et al.^26^ found that negative emotions such as disgust, anger, and sadness were more impacted by face masks when compared to happiness. However, it is important to be cautious when making comparisons between studies, since our stimuli depict emotions beyond the six basic ones as well as mental states that are not emotions at all. For example, “friendly,” “assertive,” and “relaxed,” all refer to states that are positive but distinct from “happy”. Further, we made use of different conventions for classifying mental states than those that were used in earlier studies. Our results may be interpreted as revealing a negativity bias in Theory of Mind judgements induced by the use of face masks, and this may have implications for social life.

Eye-tracking and psychophysics investigations have shown that, while the upper part of the face participates to a greater extent in expressing negative expressions, the lower part is more important for positive expressions^33–35^. It is not surprising, therefore, that obscuring the lower part of the face impairs recognition of positive mental states and decreases their perceived valence and arousal. The results of our single-case FACS analyses shed light on the facial motor patterns that are associated with the observed valence and arousal changes and provides support for the differential importance of the upper and lower face regions in the expression of negative and positive mental states respectively. The strongest AU driving the negative change in valence in the masked face, the Lip Corner Puller (AU 12), is the core component of smiling^36^. Hiding muscle activations associated with smiling likely removes a signal of positive valence in a range of facial expressions, while making muscles in the upper half of the face stand out. This may also explain why the changes in arousal show a different direction for positive and negative expressions in the first female actor: those action units that are most predictive of negative valence in the unmasked expressions—such as the Inner Brow Raiser (AU 1) and the Brow Lowerer (AU 4)^37,38^—are located in the eye region. When the lower part of the face is hidden, these muscle activations may become more salient because they are perceived in isolation. Interestingly, we found that another variable, Head Pitch (a feature of head position and therefore not affected by face masks) is significant in the interpretation of facial expression and mitigate the negative shift in ToM judgements. When the head is tilted down, the facial expression may be perceived as more negative and intense, while tilting it back does the opposite, as had been previously reported by McDuff et al.^31^. Moreover, while some of the relevant facial muscle AUs are correlated among themselves to some degree (and therefore may be hard to activate independently from each other), Head Pitch was not correlated with changes in any facial AU (see Figure S3 in the Supplementary Material). This highlights the potential compensatory mechanisms of body language to convey mental states when the face is obscured by a mask.

Our primary finding that interactions with others may be negatively colored by a distortion in ToM may have important implications for mental health and well-being. ToM is an essential socio-cognitive skill, and ToM deficits have been linked to autistic spectrum disorders, schizophrenia, decreased empathy and increased paranoid ideation^39^. Interpreting the mental states of others as more negative than they are could exacerbate existing psychiatric conditions such as social anxiety disorder and major depressive disorder^40,41^, conditions that have become markedly more prevalent in the pandemic^42^. Threatening stimuli lead to defensive reactions and involuntary decisions^43,44^; a negative distortion of ToM, even in otherwise healthy individuals, might therefore be expected to have significant psychological effects. For example, the use of masks in the classroom might hinder empathy and trust between students and teachers, thus increasing psychosocial stress during crucial developmental periods^45^.

Beyond the general shift in the phenomenology of facial expressions, the impact of masking on ToM might have other psychological consequences. For example, the mental state that was most impacted by face masks both in terms of decreased accuracy and decreased perceived arousal was “friendliness” (Fig. 2 Left and Right), while mental states like “distrustful”, “concerned” and “accusing” were among those that underwent the greatest increase in perceived arousal in Experiment 2. A reduction in the perceived friendliness of another person would not only produce a more negative experience in a perceiver but might support quite specific inferences about the motivations or intentions of others. These changes may even have clinical significance. Someone who systematically perceives other people as less friendly or benevolent, while perceiving them as more accusing or distrustful, for example, might be more vulnerable to paranoid ideation^46^. Frith^47^ proposes just such a mechanism linking negative distortions in ToM with the development of delusions in individuals with schizophrenia. Less dramatic effects might nonetheless be important; during a period of social isolation brought on by the pandemic, a systematic experience of others as less friendly and less encouraging might over time enhance feelings of loneliness and alienation, which have also been linked to negative consequences in physical and mental health (for a review, see Leigh-Hunt, 2017^48^).

While research on ToM typically construes it as a resource-intensive form of cognition, requiring deliberate, effortful processes of inference to attribute mental states to others, ToM also has to happen "on the fly"—effortlessly and quickly—to be able to anticipate action in social context and allow social interaction to proceed unimpeded^15,49,50^. Recent research shows that emotionally charged stimuli are rapidly and automatically processed^51^. Further, mental states can reliably be associated with presented pictures of eye regions in the first 100 ms after presentation, and even more rapidly for negative expressions^52^. It is possible, therefore, that the negative distortion in ToM reported here may be hard to notice and override, and this may enhance its negative impact on mental well-being.

Studying the psychosocial consequences of sustained behavior change (such as social distancing, or wearing face masks) is crucial, not only in the context of the COVID-19 pandemic, but also to be better prepared for future pandemics^53^. However valuable masks have proven to be in the prevention of the spread of infection, they may be contributing to an increase in psychological distress at a time when social support is more important than ever. Mitigating their effects—for example, by increasing awareness of their impact on social cognition, encouraging non-verbal communication beyond facial gestures (such as head tilts), or through the development and use of effective transparent masks—should be a priority.

### Limitations and future directions

Compared to most face databases, which have a large number of actors representing a limited set of emotions, our approach allows us to account for a wider range of mental states but at the expense of face diversity. In Experiments 1 and 2, all stimuli represented a single female actor and thus our results could have been directly affected by her idiosyncratic expressive patterns. To address this issue we ran Experiment 3, adding three additional sets of actors accounting for different genders (two and two male) and ethnic groups (two Caucasian, one African American and one Latino.) Even so, this is still a limited set of exemplars. Follow-up research should try to replicate these findings with a more diverse set of stimuli that would make it possible to assess the role of variables such as gender and ethnicity on changes induced by face masks. Finally, other kinds of body language, as well as contextual factors, must also be taken into account to better understand the impact of face masks in real-life social environments.

## Methods

### Participants

186 participants were recruited through public advertisements in McGill University social media forums; the first half completed Experiment 1 (n = 93; 67 female; mean age = 24 ± 7.26 SD) and the second half completed Experiment 2 (n = 93; 70 female; mean age = 23 ± 6.02 SD). An additional 100 participants, residing in Canada and in the U.S., were recruited through the website Prolific (which provides recruitment services for psychological studies) to complete Experiment 3 (48 female; mean age = 38 ± 13.1 SD). All participants had an advanced understanding of English, had normal or corrected-to-normal vision, and had never been diagnosed with a neurological condition. Prior to the experimental sessions, written informed consent was obtained from all participants, and a monetary compensation was offered in exchange for their participation. The study was approved by McGill University’s Research Ethics Board (REB approval #20-11-037). All experiments were carried out in accordance with the board’s guidelines and regulations, and in accordance with the ethical standards established by the Declaration of Helsinki.

### Stimuli

**Figure 6:**
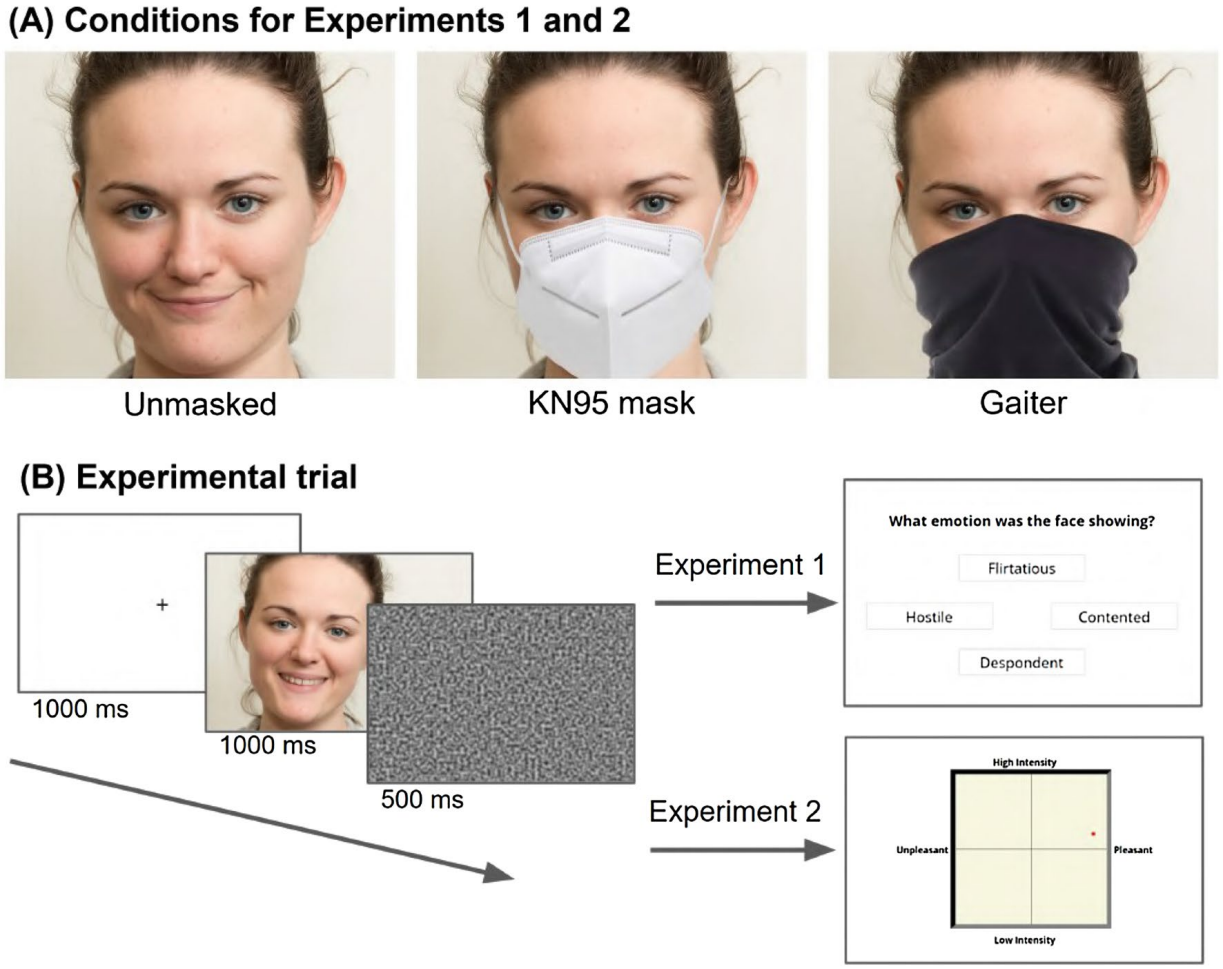
(**A**) Examples of the three conditions used in Experiments 1 and 2, which included 45 stimuli each. The KN95 mask and the neck gaiter were added to the images using Photoshop to make them look as natural as possible. Each condition was shown in a different experimental session, and each session was separated by at least a week from the previous one. (**B**) Experimental trial. For each stimulus in the condition, participants were presented with a fixation point, followed by the stimulus and then by random luminance noise. Subsequently, participants in Experiment 1 were asked to identify the mental state by selecting the correct label among four options. The three confound options presented with each stimulus were randomized, as was the spatial location in the diamond shape of each of the four labels. In Experiments 2 and 3, participants responded by clicking with their cursor in an affect grid, representing valence and arousal. There was no limit on response time in either experiment. Face images were obtained with permission from the public McGill Face Database^32^.

Stimuli for Experiments 1 and 2 were taken from the McGill Face Database^32^ and consisted of front view photographs of a female actor displaying 45 facial expressions, 32 of which correspond to the mental states evaluated in the classic "Reading the Mind in the Eyes" test (RMET)^10^. The remaining 13 expressions were those which showed the highest inter-rater agreement in the affect grid paradigm as reported in the original validation study^32^. We classified the stimuli on the basis of results obtained in a previous study^54^ in which an independent group of 95 participants assessed the unmasked facial expressions of the McGill Face Database using the same affect grid implementation used in the present study (see Fig. 6B). Facial expressions above an average score of 45 were classified as “positive”; those below − 45 were classified as “negative”, and the remaining stimuli as “neutral” (resulting in 18, 9, and 18 stimuli in each category, respectively). We note that most of the mental states in the neutral category were rated as slightly negative, a finding that was further confirmed in the present study (see the valence scores for the unmasked stimuli in Fig. 1) and which should be considered when interpreting our results.

Using Adobe Photoshop, the image of a KN95 mask was superimposed on each facial image to produce a second set of stimuli. In addition, a set of control stimuli were created by occluding roughly the same part of the face with the image of a black neck gaiter. The stimuli were thus composed of three sets of 45 facial expressions each (one unmasked, one with a KN95 mask, and one with a neck gaiter), for a total of 135 different stimuli (see Fig. 6A).

We used a more diverse set of stimuli for Experiment 3, which included pictures of both the white female actor and a white male actor taken from the McGill Face Database, as well as pictures of a black female actor and a latino male actor. The latter two were obtained from an ongoing facial expression project^55^ which follows the methodology used in the McGill Face Database. The same 45 mental states as before were selected for each actor, making a total of 180 different expressions. This experiment made use of only two conditions: unmasked faces and KN95 masks (see Fig. 3A). To account for the idiosyncratic differences in facial expression, new classifications of the 45 mental states were carried out for each actor individually based on the average scores obtained in the unmasked condition. As before, expressions above a score of 45 were classified as “positive”, those below − 45 as “negative”, and the remaining ones as “neutral”.

### Paradigm

The experiments were coded using *JsPsych*^56^. The links to the online sessions were sent via email (for Experiments 1 and 2) or via Prolific (for Experiment 3). Participants completed them on Google Chrome using their personal computers. They were asked to set their web browser zoom to 100%.

To assess both the discrete and the dimensional perspectives of mental state reading^30^, we ran Experiments 1 and 2 (n = 93 each), which differed only in the way responses were made by participants (see Fig. 6B). Both experiments consisted of three online sessions presenting one of the three sets of stimuli—unmasked faces, faces obscured by a KN95 mask, and faces obscured by a neck gaiter. The three sessions were randomized across participants and separated by at least one week, in order to avoid priming effects. To corroborate our findings across different exemplars, we ran Experiment 3 using the same paradigm as Experiment 2 but with a more diverse set of stimuli (see the Stimuli section) which included only the unmasked and KN95 mask conditions. The two sessions were also randomized across participants and separated by a week. Furthermore, participants in Experiment 3 were divided into two groups and presented with complementary sets of facial expressions, in such a way that no participant saw the same stimulus twice.

In Experiment 1, we adapted the RMET paradigm which employs a four-alternative forced-choice paradigm. Participants were shown a blank screen with a fixation point followed by the stimulus, both for 1000 ms (ms). Subsequently, a random luminance noise mask was presented for 500 ms to remove any residual visual transient. After that, participants were asked the question "What emotion was the face showing?" (the term "emotion" was used instead of "mental state" assuming that participants would be more familiar with the former than with the latter), along with four mental state terms presented in a diamond shape: the correct response together with three confounds that were randomly selected for each trial. Following the methods used to validate the stimuli^32^, and to minimize a decision bias caused by specific terms, the confounds were randomly sampled from the 93 mental states represented in the McGill Face Database. The spatial location in which the four terms appeared was also randomized.

In Experiments 2 and 3, a "point and click" task was employed^29,57^. After stimulus presentation, subjects were asked to respond by clicking with their cursor in a grid where the x-axis represents valence (measured on a scale from 150 to 150) and the y-axis represents arousal (measured on a scale from 0 to 300). In contrast with the first experiment, participants could change their responses as many times as they wanted, and were asked to press the space bar once they had made their final choice. No time limit was placed on subjects to respond in either experiment. Following the last session, participants were asked to provide demographic information and to respond to a series of questions about the pandemic.

### Statistical analysis and facial action coding system

In Experiment 1, to investigate the impact of face covering (by both the KN95 mask and the gaiter) on the recognition of mental states, we obtained three average accuracy scores (n = 93): one for positive, one for neutral, and one for negative facial expressions. Since two of these variables violated normality assumptions, we ran a Friedman (non-parametric) test for one-way repeated measures analysis of variance, considering accuracy scores for the three conditions (unmasked, KN95, and gaiter) as the dependent variables. Wilcoxon signed-rank tests with Bonferroni correction were employed as the post-hoc tests, with effect sizes computed by dividing the *Z* value by the square root of *N*.

In Experiment 2, to assess differences in valence and arousal judgements across the three conditions, we computed average valence and arousal scores for positive, neutral, and negative mental states. We then performed two series of Friedman tests: one for the valence scores, and another one for the arousal scores. Wilcoxon signed-rank tests with Bonferroni correction and effect sizes were also used for the post-hoc analyses. Since there were only two conditions in Experiment 3 (unmasked and KN95 mask), Wilcoxon signed-rank tests were used.

To assess the face muscles associated with the changes in perceived valence and arousal in Experiment 2, we used Noldus FaceReader^58^, a state-of-the-art automated analysis software for the Facial Action Coding System (FACS)^59^, to obtain the intensity scores for all of the facial action units (AU) in each of the 45 stimuli used in this experiment. The AUs are defined as individual components of muscle movement, in which one muscle, part of a muscle, or more than one muscle may be involved. McDuff et al.^31^ identified the AUs that are most associated with valenced facial expressions based on their discriminative power (defined as *P*_*i*_ = *P*(*Positive|AU*_*i*_) − *P*(*Negative|AU*_*i*_)). The AUs with the highest discriminative power (*P*_*i*_ > 0.3) were: Cheek Raiser (AU 6; *Orbicularis* *oculi*, *pars* *orbitalis*), Lip Corner Puller (AU 12; *Zygomatic* *Major*), and Lips Part (AU 25; *Depressor* *Labii*, *Relaxation* *of* *Mentalis*, *Orbicularis* *Oris*) for positive expressions; Inner Brow Raiser (AU 1; *Frontalis*, *pars* *medialis*), Brow Lowerer (AU 4; *Depressor* *Glabellae*, *Depressor* *Supercilli*, *Currugator*), Upper Lid Raiser (AU 5; *Levator* *palpebrae* *superioris*), Upper Lip Raiser (AU 10; *Levator* *Labii* *Superioris*, *Caput* *infraorbitalis*), and Head Roll for negative expressions (which was taken out of our models since it was not significant in any of them); Head Pitch for both positive and negative expressions. Note that the Nasolabial Deepener (AU 11) and Eyes Closed (AU 43) also had a high disciminative power but we could not assess them because they are not included in Noldus FaceReader. Using these nine AUs as independent variables, we ran multiple linear regressions models to identify the face muscles associated with perceived valence and arousal for both the unmasked and the KN95 stimuli. Finally, to identify the AUs most predictive of differences in scores when comparing the two conditions, we ran two other multiple linear regression models—one for changes in valence scores and one for changes in arousal scores.

## Supplementary Information

Supplementary Information.

## Data Availability

Data for all experiments are publicly available on OSF at https://osf.io/4t725/. The McGill Face Database can be accessed at http://www.gunnar-schmidtmann.com/stimuli-software.
