# Author Correction: Face masks negatively skew theory of mind judgements

**DOI:** 10.1038/s41598-023-43909-x

**Published:** 2023-10-04

**Authors:** Héctor Leos-Mendoza, Ian Gold, Fernanda Pérez-Gay Juárez

**Affiliations:** 1 Interfaculty Program of Cognitive Science, McGill University, Montreal, H3A 0G4 Canada; 2 Departments of Philosophy and Psychiatry, McGill University, Montreal, H3A 0G4 Canada

Correction to: *Scientific Reports* 10.1038/s41598-023-31680-y, published online 27 March 2023

The original version of this Article contained an error in the name of Héctor Leos-Mendoza, which was incorrectly given as Héctor Leos.

The original Article has been corrected.

