# Supplementary material for: Face masks negatively skew theory of mind judgements
Source: Sci Rep. 2023 Mar 27;13:4950. doi: 10.1038/s41598-023-31680-y (PMC10041502; doi:10.1038/s41598-023-31680-y)
Supplement: Supplementary file 1 — Supplementary Information. [file 41598_2023_31680_MOESM1_ESM.docx]

Supplementary Material


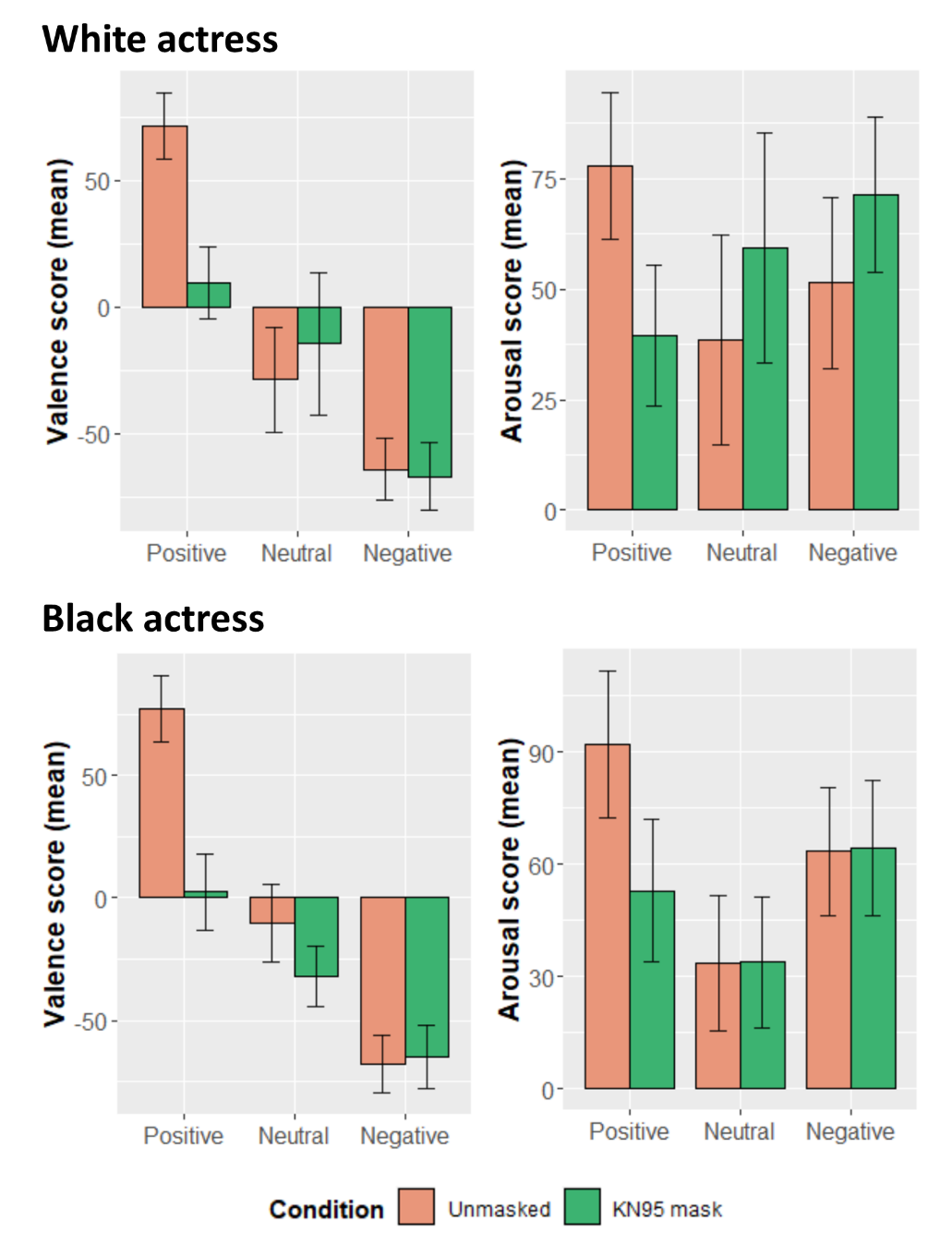


**Figure S1.** Results of Experiment 3 for each of the two female actors showing average perceived valence and arousal scores for the unmasked and the KN95 conditions. Error bars represent standard deviation of the mean.


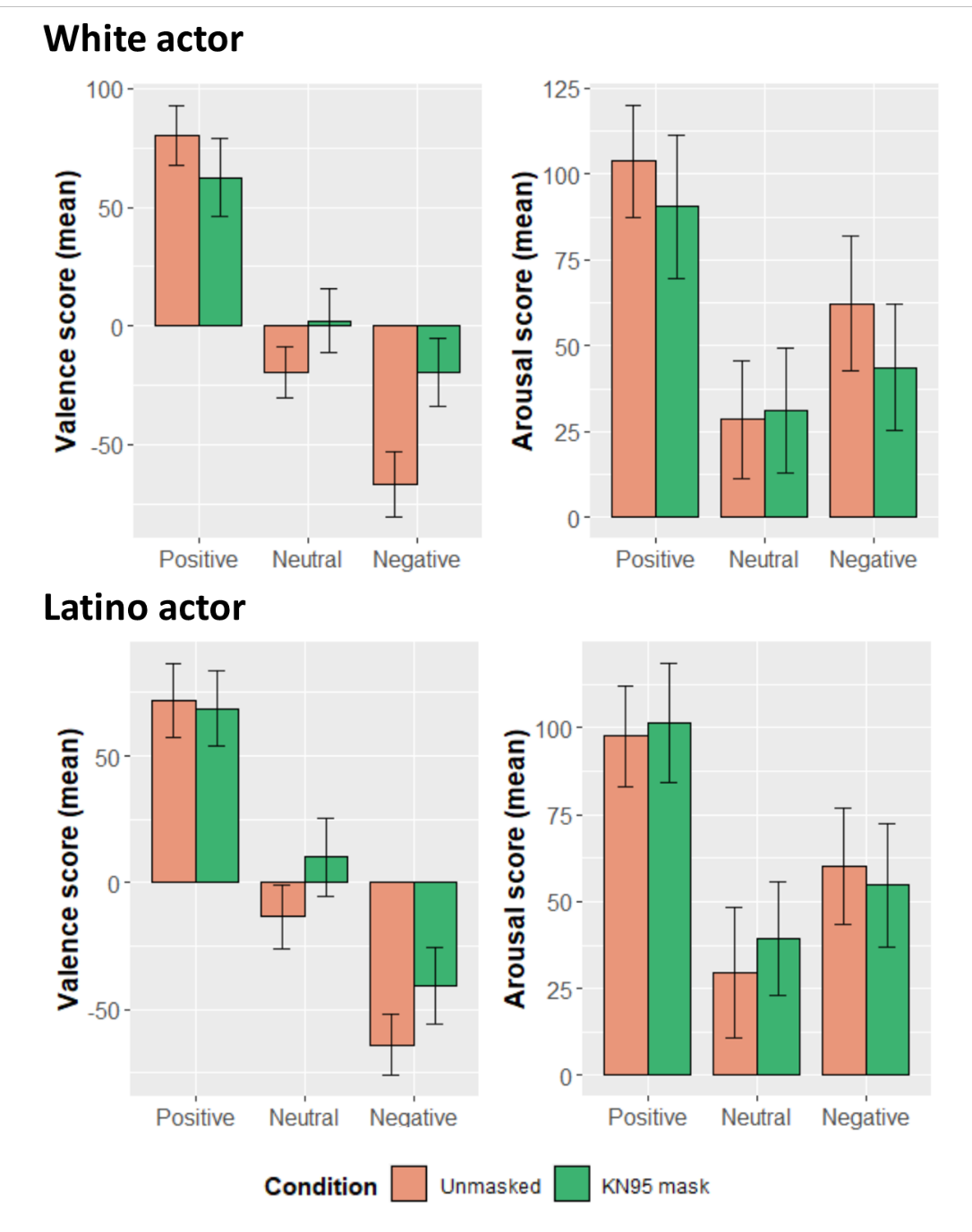


**Figure S2.** Results of Experiment 3 for each of the two male actors showing average perceived valence and arousal scores for the unmasked and the KN95 conditions. Error bars represent standard deviation of the mean.

**Table S1.** Experiment 3 statistical analyses: Wilcoxon signed-rank tests (Bonferroni corrected) were used to compare scores of the unmasked and the KN95 conditions across all actors and when looking at each actor separately. Effect sizes were calculated by diving the *Z* value by the square root of *N* (*N* = 100).


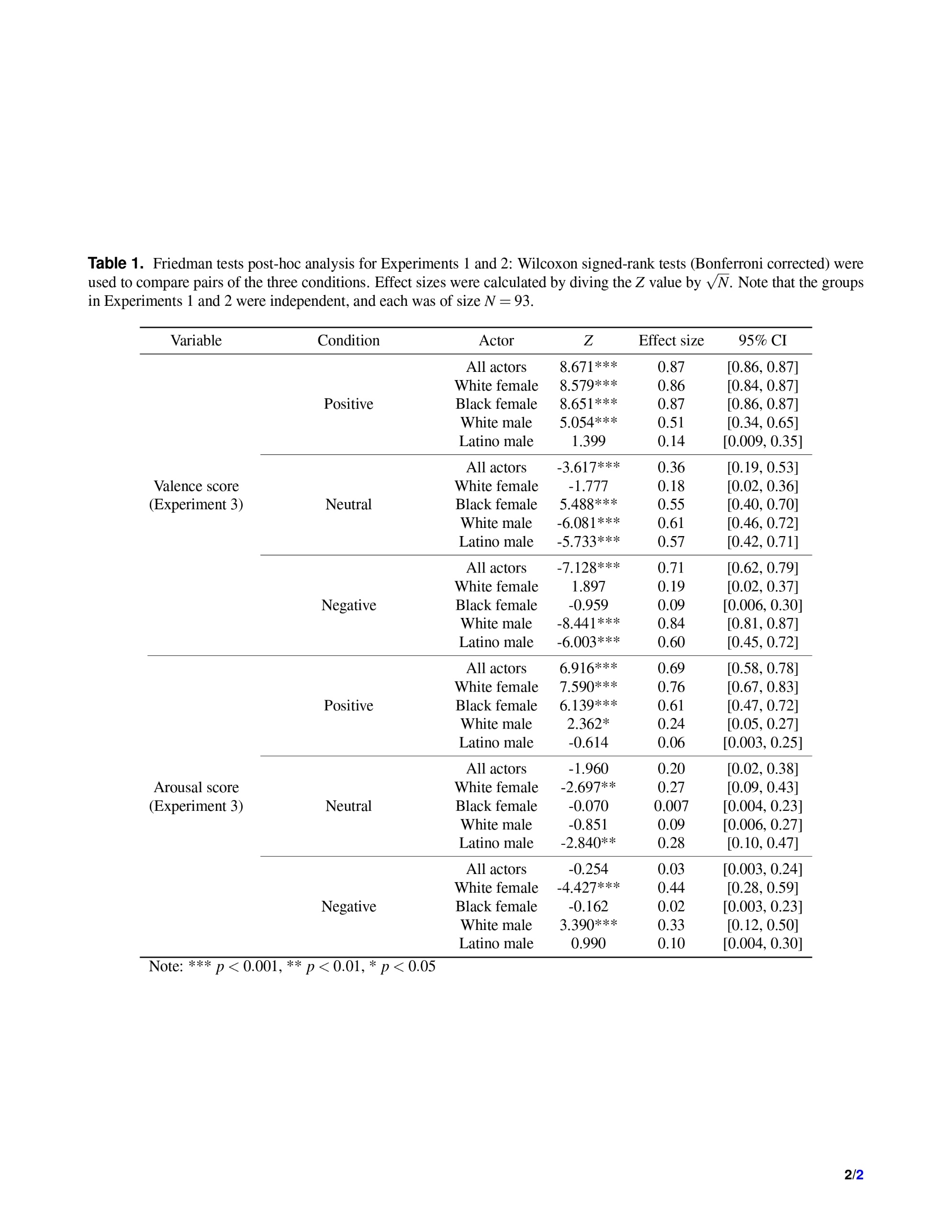


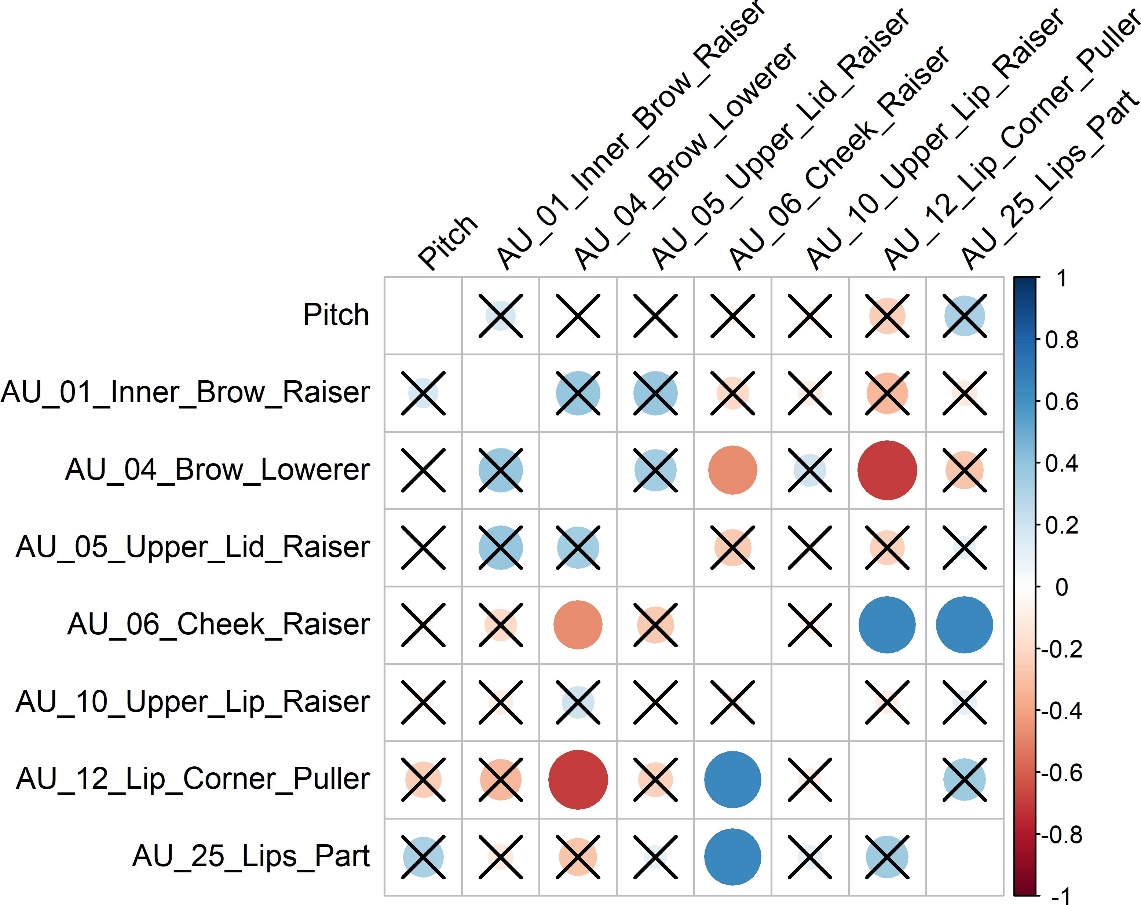


**Figure S3.** Correlations among the AUs selected for our linear regression models. Crossed-out circles represent non-significant correlations.
